# Metformin- A Promising Agent for Chemoprevention in *BRCA1* Carriers

**DOI:** 10.4172/2161-1041.1000104

**Published:** 2012-01-28

**Authors:** Nagi B. Kumar, Susan T. Vadaparampil, Nupam Mahajan, Howard S. Lilienfeld, Ji-Hyun Lee, Christine Laronga, Ardeshir Hakam, John J. Hein, Kathleen M Egan, Banu Arun, Tuya Pal

**Affiliations:** 1 Departments of Cancer Epidemiology The University of Texas MD Anderson Cancer Center, Houston, Texas; 2 Health Outcomes and Behavior, The University of Texas MD Anderson Cancer Center, Houston, Texas; 3 Molecular Biology, The University of Texas MD Anderson Cancer Center, Houston, Texas; 4 Genetics, The University of Texas MD Anderson Cancer Center, Houston, Texas; 5 Breast Cancer, The University of Texas MD Anderson Cancer Center, Houston, Texas; 6 Pathology, at the H. Lee Moffitt Cancer Center & Research Institute; 7 Oncological Sciences University of South Florida College of Medicine, Tampa Florida; 8 Breast Medical Oncology and Clinical Cancer Prevention, and Co-Director of Clinical Cancer Genetics, The University of Texas MD Anderson Cancer Center, Houston, Texas

## Introduction

Women who carry germline mutations in the *BRCA1* gene (*BRCA1* carriers) have the highest individual lifetime risk for breast cancer (BCa) known [1-3], with 50% of carriers developing breast cancer by age 50. *BRCA2* carriers also have a higher risk but relatively lower than *BRCA1* carriers. Currently available options for both *BRCA1* and *BRCA2* carriers include high-risk surveillance, risk reducing mastectomy or chemoprevention with the ant-estrogens Tamoxifen (TAM) and Raloxifene. Chemoprevention has been controversial in that *BRCA1* carriers tend to make estrogen receptor negative tumors. Selective estrogen receptor modulators (SERMs) such as tamoxifen and raloxifene reduce the risk of estrogen receptor positive breast cancers. For example, results from the National Surgical Adjuvant Breast and Bowel Project (NSABP1) Tamoxifen chemoprevention study suggested that TAM is not effective in carriers of a *BRCA1* mutation [4]. Regardless, there is conflicting evidence on this point and both TAM and Raloxifene are offered to *BRCA1* carriers as well as *BRCA2* carriers [5]. As a chemoprotective agent in the general population, Raloxifene shows a similar reduction in risk for invasive breast cancer to TAM, but not for *in situ* cancers, which comprise >20% of newly diagnosed cases; the incidence of noninvasive breast cancer is approximately 40% lower for women on TAM compared with Raloxifene [6,7]. Both TAM and Raloxifene are effective only against estrogen receptor-positive tumors [8], and Raloxifene is typically only prescribed in women who are postmenopausal and have decreased bone density [9]. Both drugs increase risk for serious side effects, including venous thrombosis and pulmonary embolism [10,11]. TAM is also associated with an increased risk for endometrial cancer and stroke [12-14]. Other side effects of both drugs include dyspareunia, cataracts, musculoskeletal complaints including leg cramps, weight gain, hot flashes, vaginal discharge, bone loss in premenopausal women and bladder control problems [15]. More recently, long-term administration of TAM was observed to cause hepatic tumors in rats, induced via a genotoxic mechanism [16]. Compounding the unfavorable side affect profile studies have determined that approximately 5 – 10% of the population carries a homozygous variant of the CYP2D6 gene that imparts low activity to convert TAM from its less active form to its active metabolite [17]. This research led the FDA to require a change in the labeling of Tamoxifen to include this information [18]. Concerns about the risk: benefit ratio have thus limited the use of TAM for prevention. Recent evidence suggests that only approximately 8.4% of BRCA carriers who have not undergone prophylactic mastectomy and are eligible to take Tamoxifen or Raloxifene [19], for risk reduction do so [20]. Certainly, there is an urgent need to identify other agents for breast cancer prevention in this high-risk group. In fact, data from our own Inherited Cancer Registry (ICARE) at Moffitt indicated that of 253 female *BRCA1* and *BRCA2* mutation carriers, 127 had remaining at risk breast tissue (including 40 with a prior breast cancer diagnosis). Of these women, 18.1% indicated that they had taken either TAM or Raloxifene (including 7/60 (11.7%) of *BRCA1* carriers and 16/67 (23.9%) of *BRCA2* carriers. Consequently, the poor uptake of existing breast cancer prevention options, which appears to be more marked in those with *BRCA1* compared to BRCA2, serves to illustrate the urgent need to identify other agents for breast cancer prevention in this high-risk group.

## Metformin and Breast Cancer Prevention in *BRCA1* Carriers

### Evidence from population and clinical studies

MET belongs to a biguanide class of oral hypoglycemic agents and is currently prescribed to over 120 million Type II diabetic patients worldwide [12], with an excellent safety profile. Recently published population studies suggest that MET decreases the incidence of cancer and cancer-related mortality in diabetic patients [7-9]. Other clinical and epidemiologic evidence links hyperinsulinemia and insulin resistance to increased mitogenic effects and thus to an increased risk of several cancers [10,11,21,22], as well as poor breast cancer outcomes [23,24]. In addition, hypothetically insulin can promote tumorigenesis via a direct effect on epithelial tissues, or indirectly by affecting the levels of other modulators, such as insulin-like growth factors, sex hormones, and adipokinesis [10,11,25]. In a more recent retrospective study of patients who received neoadjuvant chemotherapy for breast cancer showed that diabetic cancer patients receiving concomitant MET during their neoadjuvant chemotherapy had a higher pathological complete response rate than diabetic patients not receiving MET (24% versus 8%, p = 0.007) [26], demonstrating the role of MET as an antineoplastic agent for breast cancer.

### Evidence from in vitro and preclinical studies

The antineoplastic effects of MET in breast cancer are supported by a biological rationale involving important factors associated with breast cancer prognosis. Several lines of evidence have demonstrated that MET inhibits the growth of tumor cells, including breast cancer cells [20,27]. Mechanisms of action involve several pathways. In the liver, MET inhibits transcription of key gluconeogenesis genes and increases glucose uptake in skeletal muscle. Thereby reducing levels of circulating glucose, increasing insulin sensitivity, and reducing insulin resistance-associated hyperinsulinemia [28]. At the level of cell signaling, several mechanisms of MET action have been proposed; the most important one relates to the activation of AMPK [19,23,24,29-33]. MET regulates the AMPK/mTOR pathway which is implicated in the control of protein synthesis and cell proliferation. Work by Zakikhani, et al. demonstrated that MET inhibits the growth of breast cancer cells in an AMPK-dependent manner [15]. Several tumor suppressors are involved in the AMPK signaling network [15], and activated AMPK results in suppression of cell proliferation in normal and tumor cells in both *in vitro* [15], and *in vivo* studies [33]. The growth inhibition was associated with decreased mTOR activation and a general decrease in mRNA translation [19]. These observations suggest that drugs which activate AMPK may be useful in preventing cancer. Other work suggests that the affects of AMPK activation in tumor suppression are much broader than inhibition of translation, and include affects on both lipogenesis (and insulin sensitivity) and cell cycle progression [15-17]. AMPK has also been shown to affect apoptosis, with complex effects; it appears that AMPK activation may be pro-apoptotic in cells destined for malignancy [34].

The multiple signaling pathways activated by AMPK feature elements that are specifically relevant to *BRCA1*-associated breast tumorigenesis, including involvement of acetyl coenzyme A carboxylase alpha (ACCA) [16], p53 [17] and PTEN [16]. AMPK exerts its functions, at least in part, by specifically regulating the phosphorylation/dephosphorylation cycles of ACCA [35-37]. The fact that AMPK like *BRCA1*, also inactivates ACCA suggests a mechanism by which MET might substitute for loss of *BRCA1* tumor suppressive function. *In vitro* studies using an AMPK activator appeared to mimic a low energy status of the cells with increased AMPK activity that increased phosphorylation of ACCA and markedly decreased endogenous lipogenesis. Cancer cells stopped proliferating and lost their invasive properties and their ability to form colonies. *In vivo*, the chronic whole body administration of an AMPK activator attenuated the growth of human breast cancer xenografts in nude mice [32,33]. Taken together, these findings provide a molecular rationale to exploit (directly or indirectly) ACCA as a target for breast cancer prevention and/or tumor growth retardation in women with inherited mutations in *BRCA1*. AMPK is important in regulating not only lipid synthesis, but other key components required for cell proliferation, including protein and DNA synthesis [37]. Two of the most important tumor suppressors known are involved in regulation of these processes, p53 and PTEN, and both are known to play important roles in *BRCA1*-related breast cancer.

Evidence that the p53 gene pathway and BRCA genes are functionally interrelated includes the physical association of their proteins and their cooperative roles in WAF1 [21,22,38] and Bax genes transcription [21]. Additionally, somatic mutations are found at a high rate in breast cancers in *BRCA1* carriers compared with sporadic breast cancer [21], such that p53 deficiency is considered a hallmark of *BRCA1* breast tumors. *BRCA1*-associated breast tumors are associated with a unique type of p53 mutant that acquires transforming ability despite retaining a phenotype close to that of the wild-type protein in other aspects [21]. The occurrence of these mutants implies their selection specifically in the BRCA tumor-associated genetic background. Importantly, MET-induced suppression of tumor cell proliferation through activation of AMPK has been shown in xenograft mouse models to occur selectively in p53 deficient tumors [21]. Thus, MET is expected to be selectively toxic to p53-deficient cells such as those characteristic of early stages of *BRCA1* oncogenesis [21].

**Figure 1:**
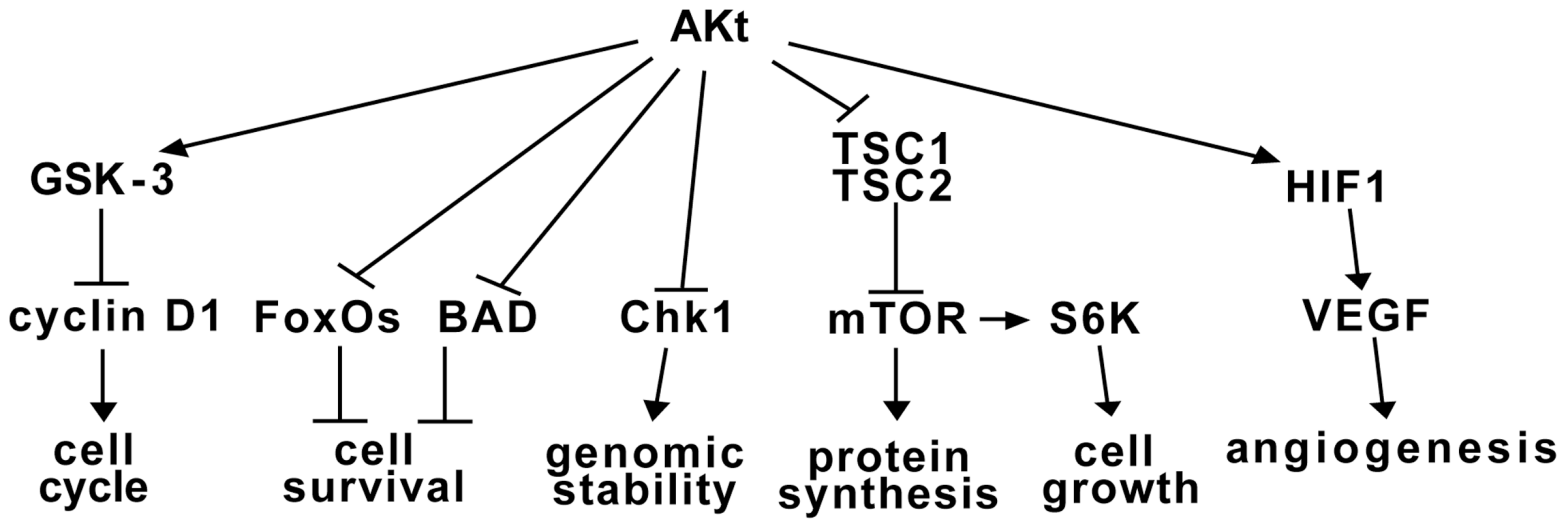
Effects of Akt Activation.

PTEN is an important tumor suppressor in breast tissue and its interaction with p53 is important in oncogenesis [21]. The PTEN promoter has a p53 binding site, and induction of p53 protein increases PTEN levels. At the same time, a positive feedback loop causes PTEN to increase p53 levels through mdm2 [21]. Thus, if a cell loses one of these tumor suppressor genes, there will be decreased levels of the other protein-one genetic hit leads to decreased activity of two important tumor suppressors. PTEN loss has also been shown to decrease expression of Rad51, a DNA repair protein that interacts with the *BRCA1* protein in double strand repair, thus enhancing tumor-related genomic instability [21]. PTEN is the critical tumor suppressor of the PI3K/Akt/mTOR signaling pathway [39]. Activation of Akt activates this potent oncogenic signaling cascade (summarized in Figure 1 below) that promotes cell transformation, proliferation, migration, angiogenesis and genomic instability; inhibits apoptosis; and maintains stem cell compartments.

In addition to activation of Akt, PTEN loss appears to inactivate feedback loops that would prevent excessive signaling through the PI3K pathway that promotes cell proliferation [40]. PTEN loss has recently been proposed as a fundamental component of BRCA-related breast tumorigenesis [31]. Current data suggest that the unique type of p53 mutations seen in *BRCA1* carriers (described above) occur in a progenitor cell prior to loss of the second *BRCA1* allele, which is known to otherwise be lethal to cells, and that the subsequent *BRCA1*-dependent DSB repair defect precipitates genetic disruption of PTEN, which is then clonally selected. This model implies that BRCA-related tumors may be addicted to aberrant PTEN-PI3K pathway signaling [26]. Importantly, activation of AMPK appears capable of overriding aberrant PTEN-PI3K pathway signaling [41,42].

### Preliminary Studies by our group

We recently performed chemosensitivity assays in the BRCA-deficient human breast cancer cell line HCC1937 in order to further assess the specific antiproliferative potential of MET relevant to *BRCA1* deficiency. Briefly, HCC1937 cells were plated 2500 cells per well in 96-well plates and treated with a series of concentrations of MET for 72h at 37°C. Subsequently, the cells were incubated with MTT at 5 mg/ml (Sigma) for 1h at 37°C and analyzed. Three independent experiments were performed. The 50% inhibitory concentration (EC50) was derived by interpolate plot analysis of the logarithmic scalar concentration curve. The results demonstrate chemosensitivity of the *BRCA*-deficient human breast cancer cell lines at similar concentrations to effects observed in other reported human breast cancer cell lines.

**Figure 2:**
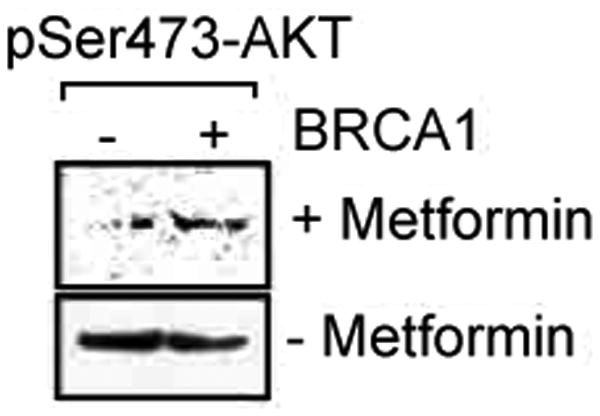
Suppression of growth of *BRCA1*-deficient breast tumors by MET.

Mutations on the *BRCA1* gene or down regulation of *BRCA1* expression activate the AKT oncogenic pathway. Indeed, the mTOR inhibitor Palomid 529, significantly suppressed *BRCA1*-deficient tumor growth in mice through inhibition of both AKT and mTOR signaling. Collectively these data indicate that activation of AKT/mTOR pathway is involved in *BRCA*1-deficiency mediated tumorigenesis and that the inhibition of AKT/mTOR pathway can be used as a target for treatment of *BRCA1*-deficient breast cancers. Elevated AMP/ATP ratio activates AMPK, which inhibits energy consuming processes and activates energy-producing processes to restore the energy homeostasis inside the cell. AMPK activators MET may inhibit breast tumorigenesis through suppression of mTOR. To test this hypothesis, we treated *BRCA-1* deficient cells and *BRCA1*-deficient cells that were stably transfected with wild type *BRCA1* gene (*BRCA1*-positive cells) with MET. mTOR activates AKT by phosphorylating it at Ser473 site. Cells were treated with MET (10nM, 1 hour) and AKT Ser473 activity was monitored by immunobloting with pSer473 antibody (Cell Signaling, MA). Significant decrease in AKT Ser473 activity was noted upon MET treatment in *BRCA1*-deficient cells (Figure 2 upper panel). Notably, untreated *BRCA*-negative cells exhibited higher levels of AKT activation (Figure 2, lower panel). Taken together, these data indicate that *BRCA*-negative cells are addicted to AKT/mTOR pathway for their survival and inhibition of mTOR by MET could significantly suppress growth of *BRCA1*-deficient breast tumors.

### Evidence from Clinical Trials

In the only, recent pilot study by Berstein et al, the investigators administered a dose of 1.0-1.5 grams/day for 3 months in 6 postmenopausal women with breast cancer, three of whom were *BRCA1* carriers and demonstrated safety as well data suggesting the possibility that aromatase complex activation in *BRCA1* mutation carriers is combined with increases in both, estrogen metabolism into catecholestrogens and their inactivation by methoxylation, and that MET may affect both of these pathways [43]. Although there are no prospective studies evaluating chemopreventive agents targeting *BRCA1* carriers, due to the complexities involved in clinical trial implementation, we conducted a survey to evaluate the interest and willingness of this target population in participating in chemoprevention trials. An anonymous web-based survey was conducted using the available population of members of FORCE (Facing Our Risk of Cancer Empowered, Inc) regarding their interest in participation in chemoprevention trials targeting *BRCA1* carriers. Responses were filtered for eligibility based on *BRCA1*+ status, no prior cancer diagnosis, and no prior mastectomy. Over the 9-day survey period, responses were received from 132 eligible women. 116 (88%) were between the ages of 25 and 50 (range 21 – 61). 37 (28%) indicated that a physician had recommended Tamoxifen while only 5 (4%) reported ever taking Tamoxifen. 39% had previously undergone bilateral prophylactic salpingo-oophorectomy. 4% had been diagnosed with diabetes or pre-diabetes; of those, none were on insulin and 2% were on an oral hypoglycemic agent. Overall, 78% indicated they would consider participation versus 22% who indicated that they would not, based on potential travel cost involved. Among those willing to participate, 89% of 103 indicated that avoiding pregnancy during the study period would be acceptable; 80% of 103 indicated that undergoing a fine-needle aspirate of their breast would be acceptable and 96% reported that a blood sample every other month would be acceptable. Thus, women who are at exceptional risk for breast cancer are a highly motivated group and a large proportion is likely to participate in research for which they are eligible.

## Future Directions

**Figure 3:**
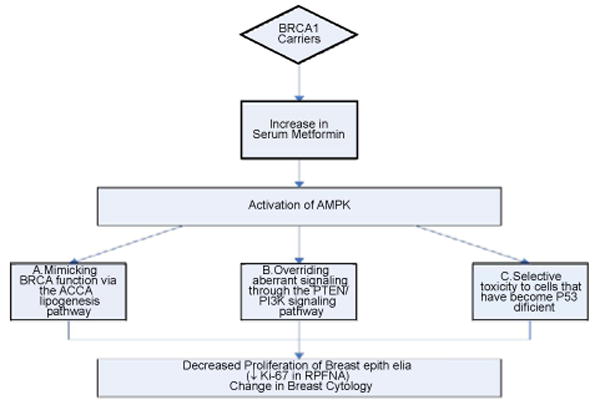
Rationale for a molecular mechanism-based approach in using Metformin for chemoprevention in BRCA1 Carriers.

Women with *BRCA1* mutations have an exceptional high risk of breast cancer and few options to reduce this risk. The choice of the AMPK activator MET appears ideally suited for chemoprevention of *BRCA1*-associated breast cancers due to: its potential to mimic *BRCA1* function in the ACCA lipogenesis pathway, including in premalignant cells; its selective toxicity to cells that have become deficient in p53, an early and hallmark event in *BRCA1*-associated breast oncogenesis and its potential to override aberrant signaling through the PTEN/PI3K signaling pathway to which *BRCA1*-associated tumors are addicted (Figure 3). The provocative results demonstrating the anticancer effects of MET in all breast cancer subtypes, including potential in *BRCA1* carriers in population studies, preclinical and retrospective clinical trials have lead to initiation of several phase I-III clinical trials evaluating MET for both for treatment and prevention in early stage to metastatic, cytotoxic therapy-resistant models of breast cancer and in adjuvant therapies. However to date, there are no clinical trials evaluating the safety and efficacy of MET in the treatment of women with *BRCA1* mutations. The current evidence that the AMPK activator- metformin appears ideally suited for chemoprevention of *BRCA1*-associated breast cancers is based on retrospective population studies and *in vitro* observations of the potential mechanism. The safety of MET has been well established. If MET can suppress proliferation in breast epithelial cells, it can therefore theoretically prevent or halt carcinogenesis in this high risk population. Future phase II clinical trials should evaluate whether changes occur in precisely selected intermediate endpoint biomarkers (IEBs) that have been identified and validated as differentially expressed in other studies of this cohort and are closely linked to the relevant pathways, in this genetic progression model for breast cancer. If such IEBs change with administration of MET, then existing knowledge of molecular targeting of MET will be enhanced. It is evident that MET has multiple properties and targets, which may be interrelated, contributing to its breast cancer prevention effects. These exploratory studies also have the potential to define novel surrogate endpoints for future clinical trials. Results of these trials have immediate benefit to the carriers themselves, but also likely to result in effective strategies for other high risk and the general population towards breast cancer prevention.
